# An antioxidation strategy based on ultra-small nanobubbles without exogenous antioxidants

**DOI:** 10.1038/s41598-023-35766-5

**Published:** 2023-05-25

**Authors:** Jin Zheng, Juncheng Qi, Sanzhao Song, Kaiwei Yuan, Lijuan Zhang, Hongwei Zhao, Junhong Lü, Beien Zhu, Yi Zhang, Jun Hu

**Affiliations:** 1 CAS Key Laboratory of Interfacial Physics and Technology, Shanghai Institute of Applied Physics, Chinese Academy of Sciences, Shanghai, 201800 China; 2 Shanghai Advanced Research Institute, Chinese Academy of Sciences, Shanghai, 201203 China; 3 University of Chinese Academy of Sciences, Beijing, 100049 China; 4 Wenzhou Institute, University of Chinese Academy of Sciences, Wenzhou, 325000 Zhejiang China

**Keywords:** Surfaces, interfaces and thin films, Nanoscience and technology

## Abstract

Antioxidation is in demand in living systems, as the excessive reactive oxygen species (ROS) in organisms lead to a variety of diseases. The conventional antioxidation strategies are mostly based on the introduction of exogenous antioxidants. However, antioxidants usually have shortcomings of poor stability, non-sustainability, and potential toxicity. Here, we proposed a novel antioxidation strategy based on ultra-small nanobubbles (NBs), in which the gas–liquid interface was employed to enrich and scavenge ROS. It was found that the ultra-small NBs (~ 10 nm) exhibited a strong inhibition on oxidization of extensive substrates by hydroxyl radicals, while the normal NBs (~ 100 nm) worked only for some substrates. Since the gas–water interface of the ultra-small NBs is non-expendable, its antioxidation would be sustainable and its effect be cumulative, which is different to that using reactive nanobubbles to eliminate free radicals as the gases are consumptive and the reaction is unsustainable. Therefore, our antioxidation strategy based on ultra-small NB would provide a new solution for antioxidation in bioscience as well as other fields such as materials, chemical industry, food industry, etc.

## Introduction

In living systems, antioxidation is one of the most concerned issues since reactive oxygen species (ROS) are usually produced persistently along with normal cellular metabolism^1,2^. However, excessive ROS often causes oxidative damage to a variety of important cellular components, including lipids, proteins, and DNA molecules^3–6^. Currently, various antioxidants have been suggested as dietary supplements to reduce ROS-associated diseases^7^. The effectiveness of those antioxidants has been proven in the treatments of many oxidative damage-caused acute diseases^8,9^. However, in recent decades, most clinical trials in the treatments of oxidative damage-caused chronic diseases by the supplements of antioxidants have not provided convincing evidence for the clinical benefits^10^. Badly, some antioxidants even have toxic side effects^11–15^, and most of them are non-sustainable in use and become unstable due to their sensitivity to normal environments^16–21^. Therefore, novel antioxidation strategies with high stability, sustainability, and biologically-safety are demanded.

Gas–liquid interface has long been recognized to have unique physical, chemical, and biochemical properties. Recently, it has been employed to regulate many oxidation/reduction reactions. Some simulations and experimental evidence have shown that gas–liquid interfaces could enrich ROS and regulate the processes of their generation and quenching^22–25^, resulting in enhancing/inhibiting the substrate oxidation reaction by ROS. For example, Heath and Valsaraj^26^ studied the process of the enrichment of ROS and the reactants at the gas–liquid interface and found that the reaction rate was largely promoted by several orders as compared with that in bulk solutions. Nam and Richard^27–29^ found that oxidation or reduction would occur at the gas–liquid interfaces of small water droplets for different kinds of substrates. In these studies, the gas–liquid interface takes effect through the adsorption of ROS and/or substrates. Thus, if the surface area of a gas–liquid interface is so small that it prefers to enrich ROS but has insufficient space for larger substances, it may exhibit a certain antioxidant activity for a series of substrates. So far, the size effects of the gas–liquid interface on the reactivity have not been investigated like that of nanodroplets^30,31^.

Nanobubbles (NBs), typically as a nanoscale gas-phase suspended in the water phase^32,33^, can provide a large number of gas–liquid interfaces that may be employed for the enrichment of ROS. The size of the NBs varies from ~ 10 nm (ultra-small NBs) to hundreds of nanometers (normal NBs); therefore, it is a suitable model to study the antioxidation or oxidation of a gas–liquid interface. Previously, it has been reported that oxygen NBs promoted the formation of ROS by producing hydroxyl radicals through the collapse of the microbubbles^34^, while the reductive hydrogen NBs helped the quenching of ROS^35,36^. However, in these studies, the chemical properties of the gas phases rather than the size of NBs were focused on, in which the gases in the nanobubbles are consumptive and would run out so that the redox reaction is unsustainable.

In this study, an antioxidation strategy based on ultra-small NBs without exogenous antioxidants was provided. A significant size dependence was observed when the NBs were employed to determine their ability to block the oxidization of substances by the hydroxyl radicals. It was found that the ultra-small NBs exhibited a strong antioxidant effect for extensive substrates, while normal NBs worked only for some substrates. Since the gas–water interface of the ultra-small NBs is non-expendable, its antioxidation would be sustainable and its effect be cumulative. We believe that this research would help develop new solutions for removing excess free radicals in a system without reductants supply.

## Results

### Antioxidation of ultra-small N_2_ NBs

**Figure 1:**
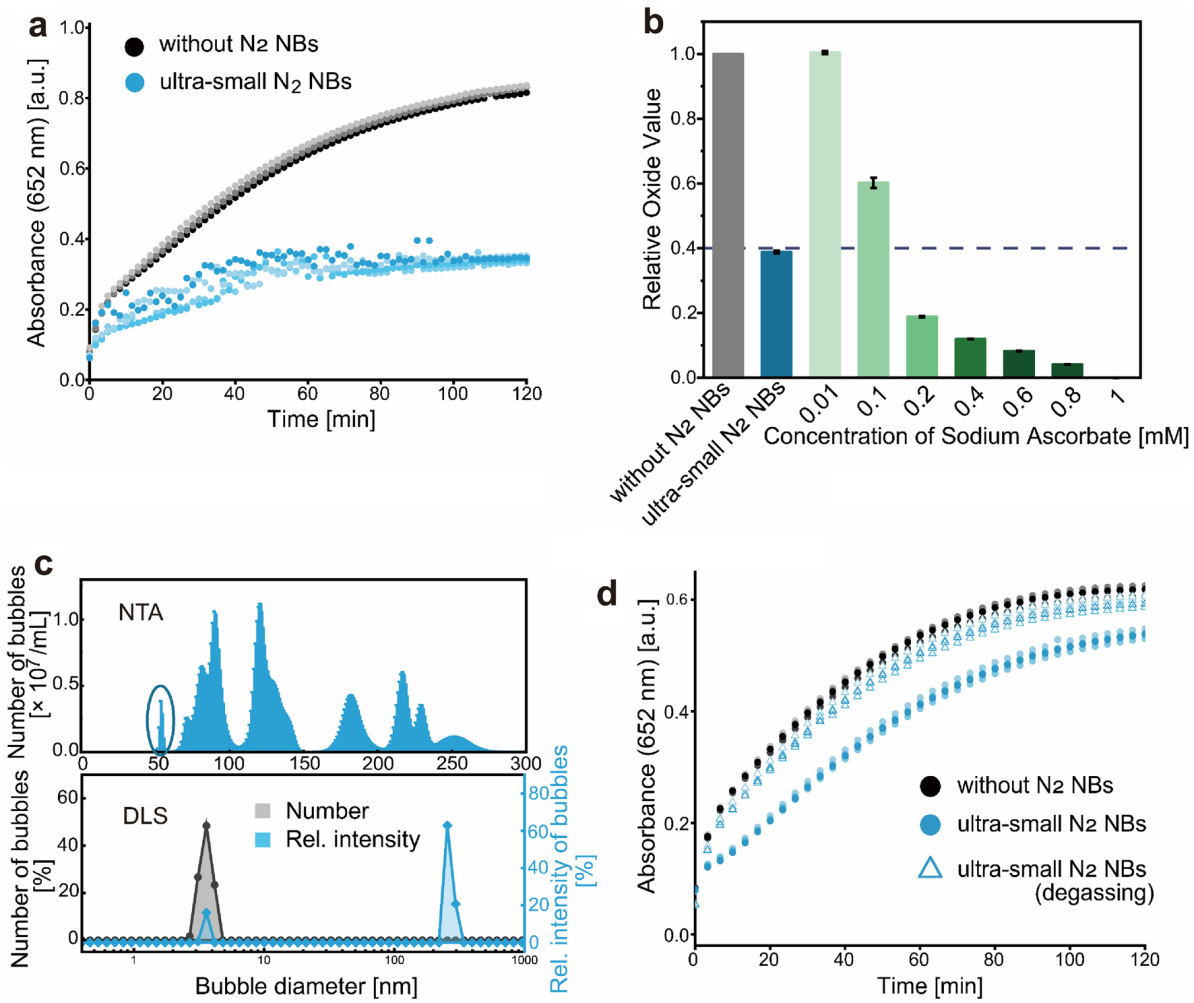
Antioxidation of ultra-small N_2_ NBs. (**a**) Oxidation curves of TMB in water containing the ultra-small N_2_ NBs. (**b**) Comparison of the relative oxide value of the ultra-small N_2_ NBs with the sodium ascorbate with different concentrations. The oxidation curves of TMB in water containing different concentrations of sodium ascorbate were firstly measured like (**a**). Then the maximum absorption value was obtained from each oxidation curve. The relative oxide value of each item was calculated with: $Relative oxide\;value\;\left( {item} \right) = \frac{{Max\;absorbance\;\left( {item} \right)}}{{Max\;absorbance\;\left( {without\;NBs} \right)}}$. (**c**) NB size distribution measured by NTA (upper) and DLS (bottom). Circles highlighted the peak of the NBs with a size of about 50 nm. (**d**) Oxidation curves of TMB in ultra-small N_2_ NBs-containing water before and after degassing.

The experiment was first conducted by determining the antioxidant effect of the ultra-small nitrogen (N_2_) NBs by detecting their ability to block the oxidization of 3, 3′, 5, 5′-tetramethylbenzidine (TMB) caused by the hydroxyl radicals (Figures S1 and S2) generated from H_2_O_2_ with the catalysis of Cu^2+^. Ultra-small N_2_ NBs were generated in cold pure water (0 °C) during a compression-decompression process^37^ and then were introduced to the oxidation-reaction system under room temperature and atmospheric pressure. The oxidation curves were obtained by monitoring the absorbance at 652 nm of the oxidized product of TMB^38^. It’s worth noting the N_2_ NB itself has no detectable absorbance at 652 nm (Figures S3), and the redox potentials of N_2_ NB-containing water were similar to that of pure water (Table S1). The results showed that the oxidation rates of TMB in water containing ultra-small N_2_ NBs were greatly reduced in comparison to that in pure water along with the increase of reaction time, and the absorbance values at plateau were much lower than that in pure water (Fig. 1a), which suggests a strong antioxidant effect of the ultra-small N_2_ NBs. In addition, a comparative study indicated that the antioxidant ability of the ultra-small N_2_ NBs was equivalent to a common antioxidant, sodium ascorbate, in a concentration between 100 and 200 μM (Fig. 1b).

In our experiments, nanoparticle tracking analysis (NTA) and dynamic light scattering (DLS) were employed as complementary means to determine the size distribution and concentration of nanobubbles in water^39^. By monitoring the Brownian Motion of a relatively small number of individual objects, NTA is able to accurately measure the concentration (10^6^–10^9^ particles/mL) and size (10–2000 nm) of polydisperse populations^40^. Due to the low light scattering of NBs in water, NTA can test their size distribution in the range of 50–2000 nm, meanwhile determining their concentration. In the case of DLS, the collective diffusion of a larger number of objects is monitored and their average size is calculated. However, DLS only provides a rough size distribution of samples ranging from 0.3 nm to 15 μm without concentration information^41,42^. Figure 1c (upper) showed a typical size distribution of the as-generated N_2_ NBs as measured by NTA, with the peaks mostly between 50 and 270 nm. NTA analysis also indicated a NB concentration of 5.42 × 10^7^ ± 5.78 × 10^6^ particle/ml and an averaged NB size of 152.7 ± 14.1 nm. Figure 1c (bottom) showed two peaks with very strong scatter intensity in the DLS curves, indicating that the sizes mostly centered at 3.62 nm and 255 nm, respectively. The only peak observed in the DLS number percent curve (Fig. 1c, bottom) centered at 3.62 nm, suggesting that these ultra-small NBs made up the overwhelming majority in numbers in the solution.

A degassing experiment was carried out to rule out the possibility that the introduction of impurities during NB generation might have also caused the observed antioxidant effect. By removing most of the N_2_ NBs in water after degassing for 24 h under a vacuum of 0.01 atm (Fig. S4), TMB oxidization curves (Fig. 1d) showed that the antioxidation ability of the N_2_ NBs water was significantly reduced, clearly confirming that the observed antioxidant effect was originated from the N_2_ NBs rather than from impurities.

### Size dependence of the N_2_ NB’s antioxidant capability

**Figure 2:**
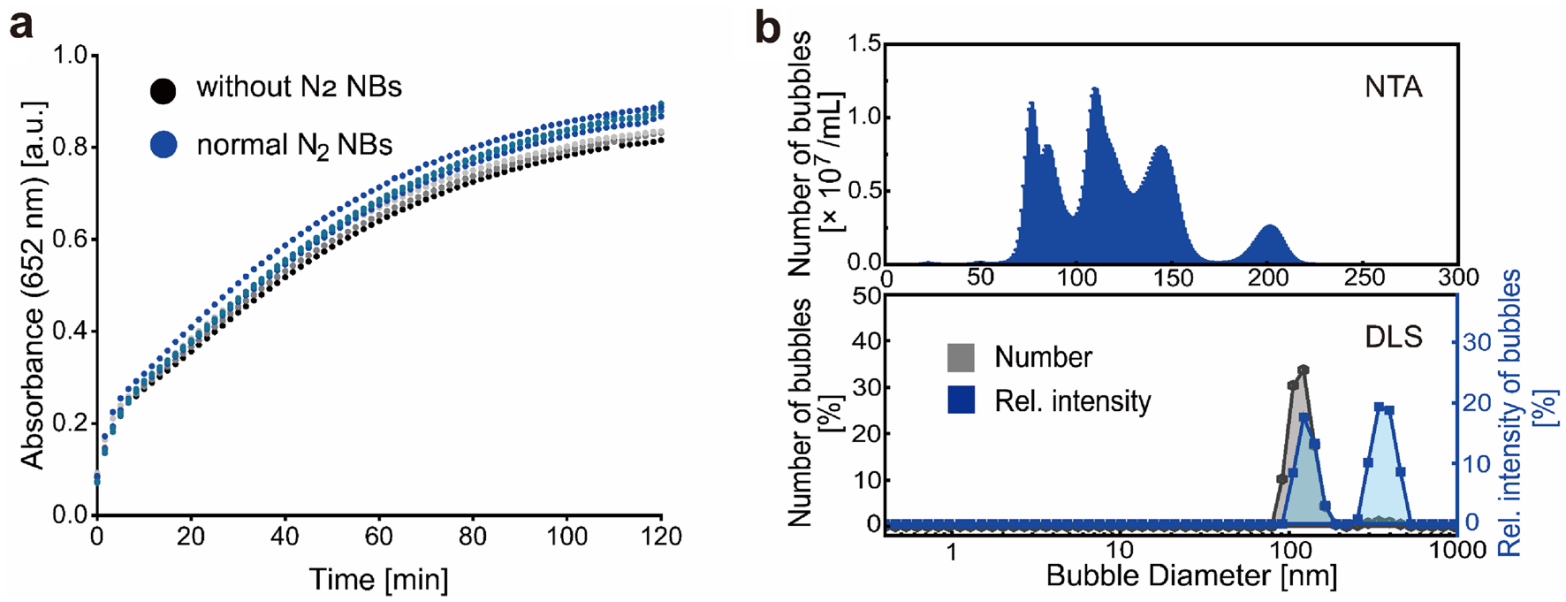
Normal N_2_ NBs-assisted oxidation of TMB. (**a**) Oxidation curves of TMB in water containing normal N_2_ NBs. (**b**) Size distribution curves of normal N_2_ NBs as measured by NTA (upper) and DLS (bottom).

Since the size of the N_2_ NBs generated was widely distributed in the range of 0–300 nm (Fig. 1c), it was plausible to explore if there would have a size dependence for their antioxidant capability. We found that the normal N_2_ NBs generated in fresh ultrapure water at room temperature did not inhibit but slightly enhance the oxidation of TMB (Fig. 2a). NTA study showed a typical size distribution of the normal N_2_ NBs between 70 and 220 nm (Fig. 2b, upper), a NB concentration of 6.41 × 10^7^ ± 1.72 × 10^7^ particle/ml, and an averaged NB size of 116.9 ± 14.7 nm. DLS study revealed two strong scattering intensity peaks centered at 142 and 396 nm, respectively (Fig. 2b, bottom). Both NTA and DLS results of normal N_2_ NBs showed no detectable NBs with sizes smaller than 50 nm, implying that the antioxidant effect was only caused by the ultra-small NBs (typically < 50 nm). Besides, we found that the ultra-small N_2_ NBs transformed from normal N_2_ NBs through a freeze-thawing operation also exhibit an antioxidant effect (Fig. S5). In addition to the ultra-small N_2_ NBs, ultra-small oxygen (O_2_) NBs also have a strong antioxidant effect in the TMB oxidation reaction (Fig. S6).

### The antioxidation mechanism for the ultra-small NBs

**Figure 3:**
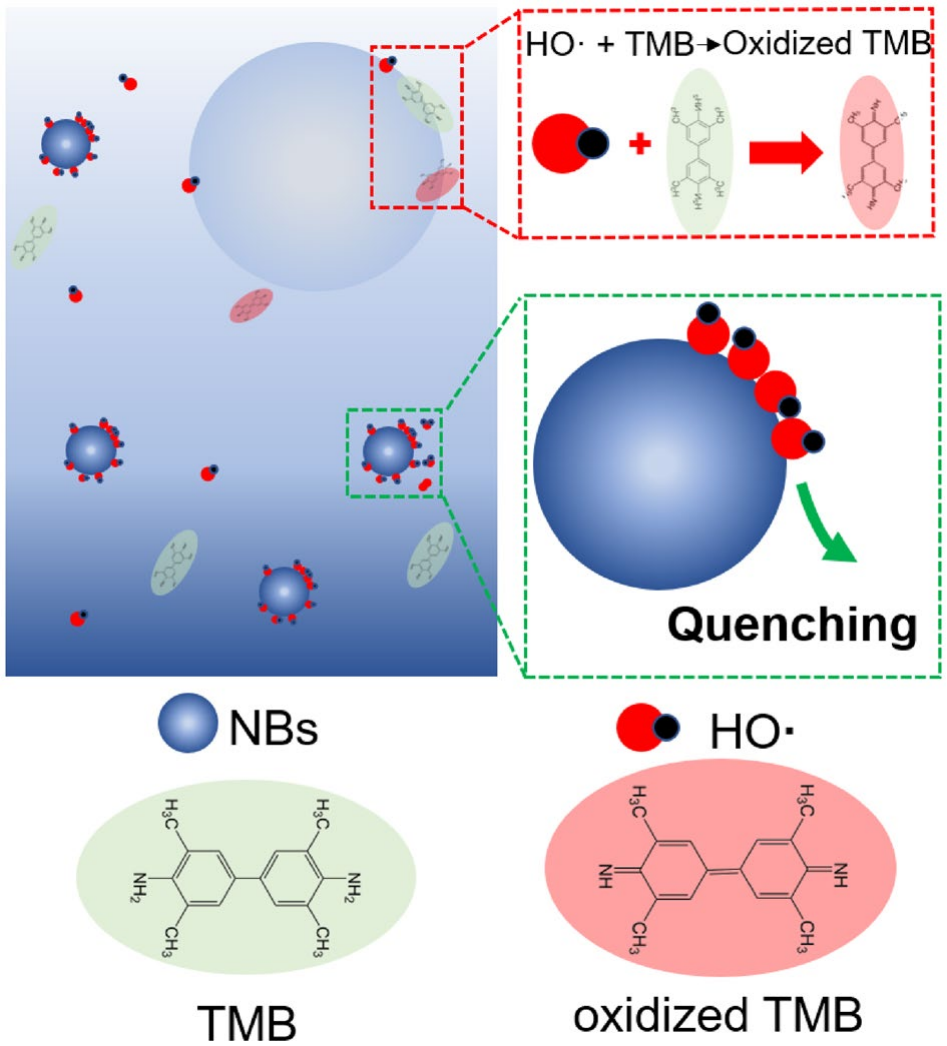
The mechanism of antioxidation of the ultra-small NBs.

The above results clearly showed that there was a size dependence on the NB’s antioxidant capability. Ultra-small N_2_ NBs inhibited the oxidization of TMB by hydroxyl radicals, while their clusters or normal N_2_ NBs (typically > 50 nm) slightly enhance the oxidation of TMB. The contrasting effects of the small and large NBs on the TMB oxidation seemed difficult to be understood. Currently, our knowledge about the chemical properties of the interfaces of NBs is much poor, it is wise to interpret our observations based on the existing realizations regarding the regulation of oxidation and reduction by gas–water interfaces. Since the electrical surface potential difference of NBs is normally − 20 mV, far smaller than the 3 V at the gas–liquid interface of small water droplets^28,43^. Thus, it is not appropriate to explain our results from the electrical surface field mechanism proposed by Nam and Richard. Previous studies have shown that, when free radicals and substrates were both enriched at the gas–liquid interfaces, the oxidizing reaction could be accelerated^26,44^. Therefore, we believed that the selective enrichment of ROS at the gas–liquid interface of the NBs might play an important role in our reaction systems. A plausible explanation may be that the surface areas of the ultra-small NBs were so small and had insufficient space for larger substrate molecules to be easily adsorbed, which resulted in the fact that it preferred to enrich more ROS but fewer substrate molecules. The short-lifetime hydroxyl radicals would be enriched at the interface and quenched by themselves (Fig. 3). In contrast, the big surface area of the large NBs (or NB clusters) would enrich both the TMB and the hydroxyl radicals at their gas–liquid interfaces, and enhance the reaction between TMB and hydroxyl radicals as usual. This mechanism also works for another classic hydroxyl radical probe, 2,2'-Azinobis-(3-ethylbenzthiazoline-6-sulphonate) (ABTS) (Fig. S7). In addition to the hydroxyl radicals, the ultra-small NBs were also found to scavenge superoxide anion radicals (Fig. S8).

### Antioxidation of N_2_ NBs for hydrophilic substrates

**Figure 4:**
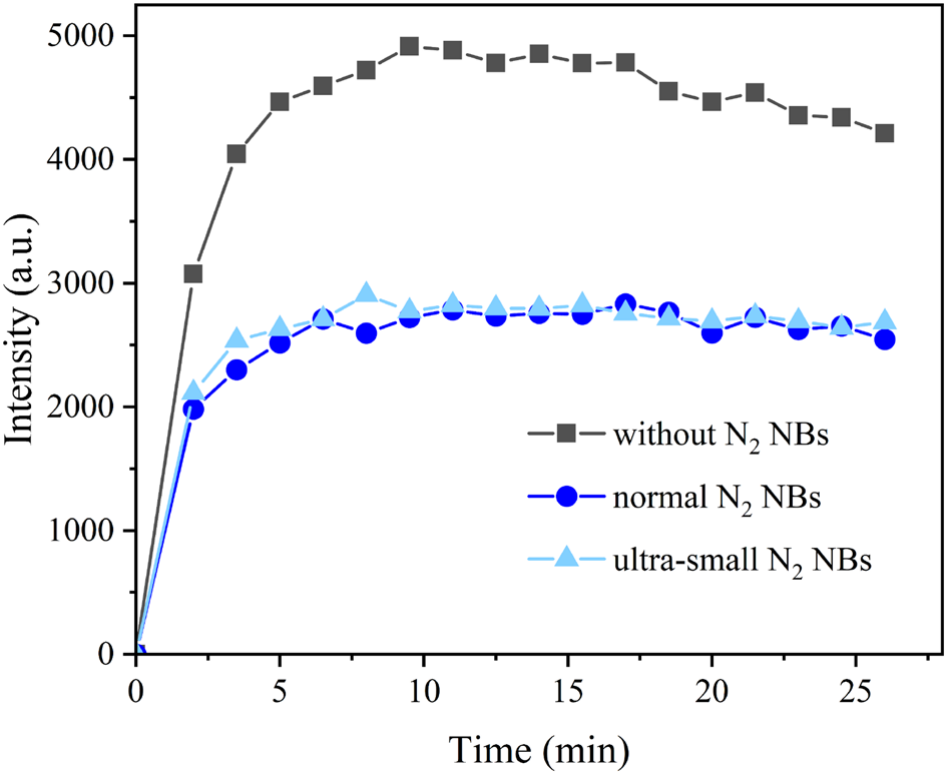
The DMPO-OH signal intensity in water containing normal N_2_ NBs or ultra-small N_2_ NBs over time.

According to our proposed mechanism (Fig. 3), normal NBs enhance oxidation due to that they simultaneously adsorb ROS and hydrophobic TMB at the gas–liquid interface, which increases their reaction probability. If this is the case, normal NBs should also exhibit antioxidant effects when substrate molecules that tend to remain in the water phase rather than at the gas–liquid interface are used. To test this hypothesis, dimethyl pyridine N-oxide (DMPO), a commonly-used electron spin-resonance (ESR) spin trap, was employed for capturing hydroxyl radicals. DMPO is hydrophilic so that it should present in the water phase. In this experiment, ESR was used to measure the intensity of the oxidized DMPO (DMPO-OH). Results (Fig. 4) showed that DMPO-OH signals in reaction systems containing normal N_2_ NBs or ultra-small N_2_ NBs were much lower than that of the control group, indicating an antioxidation effect. The results further support our mechanism.

## Discussion

**Figure 5:**
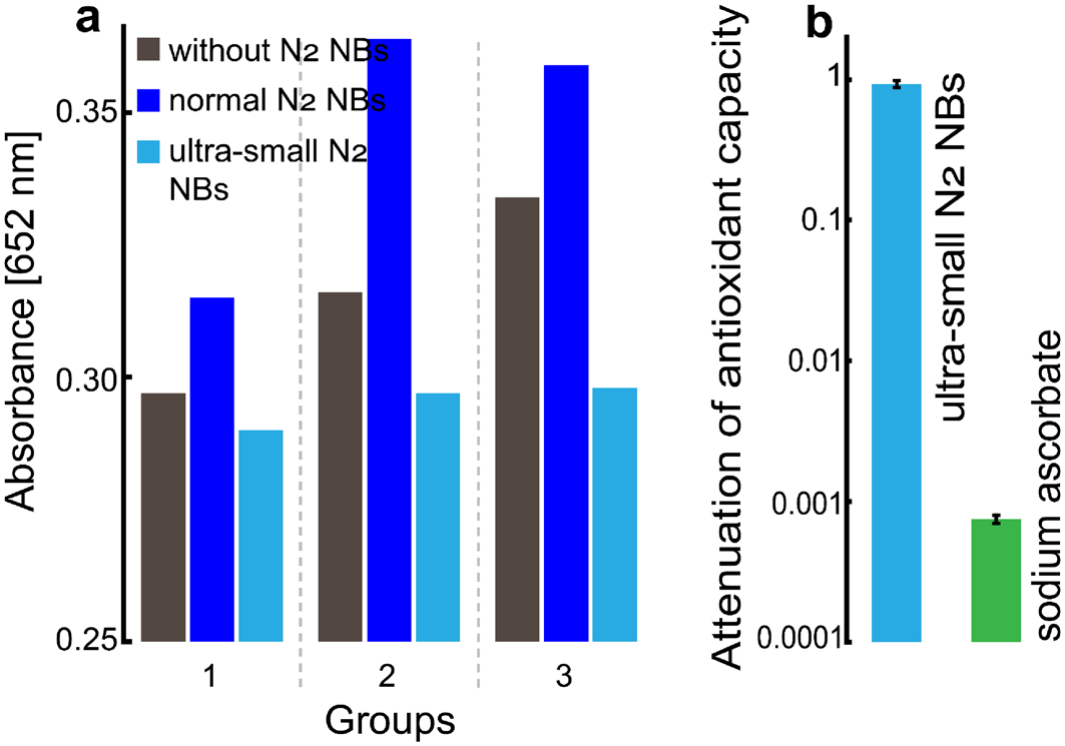
The absorbance of the oxidized TMB by the hydroxyl radicals generated by UV radiation. (**a**) The absorbance of the oxidized TMB in three parallel groups after UV radiation. (**b**) The attenuation of the antioxidant capacity of the ultra-small N_2_ NBs and 1 mM sodium ascorbate after UV radiation for 3 h.

To exclude the possible involvement of the reaction system in the antioxidation of the NBs, we employed ultraviolet (UV) radiation^45–47^ (Figs. 5a and S9) or Fe^2+^ instead of Cu^2+^ (Fig. S10) to produce hydroxyl radicals from H_2_O_2_. Results also showed a strong antioxidant effect of the ultra-small N_2_ NBs in contrast to a slight pro-oxidant effect of the normal N_2_ NBs. Thus, we believe that the antioxidant effect of the reductant-free NBs should be mainly ascribed to the ultra-small NBs themself.

Compared with conventional reducing agents including reactive nanobubbles^48,49^ that are consumptive in the reaction, the use of ultra-small NBs as an antioxidant has certain advantages. First, the ultra-small NBs are stable in pure water^41^ and their antioxidant ability could be sustained even under a high-level ROS environment. For example, the ultra-small N_2_ NBs kept almost 100% of their antioxidant ability (Fig. 5b) in a system containing high-level hydroxyl radicals that were constantly generated by intense UV radiation of H_2_O_2_ in water. In contrast, 1 mM sodium ascorbate was consumed gradually and only kept ~ 0.1% of its original antioxidant ability (Fig. 5b) under the same condition. Second, according to the proposed antioxidation mechanism, there would be no harmful oxidized products remaining after quenching ROS with the ultra-small NBs. We believe that this unique property is essentially important in the application of ultra-small NBs as an antioxidant in living systems. The high-dose exogenous antioxidants and oxidation products are often harmful to normal cells^14,50^. Third, ultra-small NBs are stable and can have a persistent antioxidant function, while many antioxidants are susceptible to the environment and would degrade in storage and transportation. It should be noted that, in principle, the ultra-small NBs are only effective to scavenge short-lifetime ROS but hard to remove long-lifetime free radicals. Fortunately, most of the harmful radicals produced in living organisms are short life-time ROS.

## Conclusion

In summary, an antioxidant effect of ultra-small NBs has been explored. Our results indicated that the ultra-small NBs had an obvious effect to inhibit the oxidation of hydrophobic substrates (TMB) or hydrophilic substrates (DMPO) caused by hydroxyl radicals. Since there was no special chemical reducing agents added in the reaction system, the antioxidation ability of the ultra-small NBs could be used safely in living systems and might find its potential applications in relieving oxidative stress in organisms including human beings. In addition, our results may also provide a new scientific view to the controversial issue about the claimed healthy effects of some natural or ‘functional’ water^51^, since NBs are believed to exist ubiquitously in nature. Further explorations should be conducted in developing techniques to prepare ultra-small NBs with higher concentrations and more precise regulation of the size distributions to fit the antioxidation demands in many practical applications.

## Materials and methods

### Materials

Ultrapure water was prepared from an ELGA LabWater (ELGA Classic-PURELAB) instrument. Copper(II) chloride dihydrate (analytical grade, ≥ 99%, Sinopharm Chemical Reagent Co., Ltd) and 3, 3′, 5, 5′-Tetramethylbenzidine (analytical grade, ≥ 99%, Macklin Reagent); 2,2′-Azino-bis(3-ethylbenzothiazoline-6-sulfonic acid) diammonium salt (≥ 98%, OKA); Iron(II) sulfate heptahydrate (analytical grade, ≥ 99%, Sinopharm Chemical Reagent Co., Ltd); 1,2,3-Trihydroxybenzene (analytical grade, Sinopharm Chemical Reagent Co., Ltd); Hydrogen peroxide 30% aqueous solution (in guaranteed grade, Sinopharm Chemical Reagent Co., Ltd); 5,5-Dimethyl-1-Pyrrolidine-N-oxide (≥ 98%, SIAL); The purity of nitrogen and oxygen is greater than 99.999%. These chemicals were used as received without further purification.

### Generation of NBs

The NBs were generated by a compression-decompression method in ultrapure water that was previously reported^37^. The experiment was carried out in a custom-made metal chamber with pressure control. First, ultrapure water was placed into the chamber, and gas (N_2_ or O_2_) was introduced into the chamber to a pressure of 0.6 MPa. Then, the pressure in the chamber was slowly reduced (20 sccm) to normal pressure (1 atm). NBs were generated in ultrapure water that was either at room temperature or at 0 °C that was prepared from a mixture of ice and water.

### Analysis of NBs

The nanoparticle tracking analysis (NTA) system (NS300, Malvern, UK) was used to analyze the number density and size of the prepared NBs in water. NTA 3.4 software was used to capture and analyze data. Besides, a dynamic light scattering (DLS, nano-ZS90, Malvern) instrument was also employed to detect the scattering light intensity and number (%) of NBs.

### Determination of TMB oxidation curve

The antioxidant effect of N_2_ NBs was determined by the ability to block the oxidization of TMB (final concentration of 0.4 mM) by the hydroxyl radicals that were generated from H_2_O_2_ (final concentration of 0.8 M) with the catalysis of Cu^2+^ (10 μM). The effect of NBs on the oxidation kinetics of TMB was determined by monitoring the absorbance at 652 nm. The final reaction volume was set to 1 ml by adding ultrapure/NBs water. After preparing the reaction mixtures, they were immediately transferred to a 96-well plate (each sample of 200 μL in 4 wells). A microplate reader (VERSA max microplate reader) was employed to monitor the optical density change at 652 nm.

### Determination of TMB oxidation curve in Fe^2+^–H_2_O_2_ reaction system

Oxidation curve of TMB under normal N_2_ NBs or ultra-small N_2_ NBs in Fe^2+^/H_2_O_2_ system. The final concentrations of Fe^2+^, TMB, and H_2_O_2_ were 50 μM, 0.4 mM, and 80 mM, respectively. The final reaction volume was set to 1 ml by adding ultra-pure/NBs water. After preparing the reaction mixtures, they were immediately transferred to a 96-well plate (each sample of 200 μL in 4 wells). A microplate reader (VERSA max microplate reader) was employed to monitor the optical density change at 652 nm.

### Determination of ABTS oxidation curve

The antioxidant effect of N_2_ NBs was determined by the ability to block the oxidization of ABTS (final concentration of 200 μg/mL) by the hydroxyl radicals that were generated from H_2_O_2_ (final concentration of 0.8 M) with the catalysis of Cu^2+^ (10 μM). The effect of N_2_ NBs on the oxidation kinetics of ABTS was determined by monitoring the absorbance at 405 nm. The final reaction volume was set to 1 ml by adding ultrapure/NBs water. After preparing the reaction mixtures, they were immediately transferred to a 96-well plate (each sample of 200 μL in 4 wells). A microplate reader (VERSA max microplate reader) was employed to monitor the optical density change at 405 nm.

### Determination of auto-oxidation curve of pyrogallol

Under alkaline conditions, pyrogallol can rapidly auto-oxidize to release O_2_^−^·, and generate a colored intermediate product which that has a strong light absorption at the wavelength of 325 nm. When there is a substance capable of quenching O_2_^−^·, the accumulation of the intermediate products would be prevented. The final concentration of pyrogallol was 0.1 mM, and the buffer system was a Tris–HCl buffer solution with pH 8.0. After preparing the reaction mixtures, they were immediately transferred to a 96-well plate (each sample of 200 μL in 4 wells). A microplate reader (VERSA max microplate reader) was employed to monitor the optical density change at 325 nm.

### ESR measurement

The liquid sample was sucked into the capillary tube and sealed for ESR measurement. The measurement parameters are as follows: Centra field: 324 mT; Sweep width: 5.0 × 1 mT; Mod.freq.: 100.00 kHz; Mod. width + /−: 0.35 × 1 mT; Sweep time: 1 min. ESR Data Process software was used to analyze data.

### UV radiation

The reaction solution (2 ml) containing TMB (0.4 mM) and H_2_O_2_ (0.08 M) in a quartz cuvette (1 × 1 × 5 cm^3^) was placed 30 cm away from a UV lamp (20 W) and was irradiated at a wavelength of 256 nm for 10 min. The solution’s absorbance value at a wavelength of 652 nm was measured at the end of the radiation.

## Supplementary Information

Supplementary Information.

## Data Availability

All data generated or analysed during this study are included in this published article and its supplementary information files.
