# Supplementary material for: An antioxidation strategy based on ultra-small nanobubbles without exogenous antioxidants
Source: Sci Rep. 2023 May 25;13:8455. doi: 10.1038/s41598-023-35766-5 (PMC10212996; doi:10.1038/s41598-023-35766-5)
Supplement: Supplementary file 1 — Supplementary Information. [file 41598_2023_35766_MOESM1_ESM.docx]

Supporting Information

An antioxidation strategy based on ultra-small nanobubbles without exogenous antioxidants

Jin Zheng^1,3^*****; Juncheng Qi^1,3^*****; Sanzhao Song^4^; Kaiwei Yuan^1,3^; Lijuan Zhang^1,2^, Hongwei Zhao^1,2^, Junhong Lü^1,2^, Beien Zhu^1,2^, Yi Zhang^1,2^**^🖂^**; and Jun Hu^1,2^**^🖂^**

^1^CAS key Laboratory of Interfacial Physics and Technology, Shanghai Institute of Applied Physics, Chinese Academy of Sciences, Shanghai 201800, China.

^2^Shanghai Advanced Research Institute, Chinese Academy of Sciences, Shanghai 201203, China.

^3^University of Chinese Academy of Sciences, Beijing 100049, China.

^4^Wenzhou Institute, University of Chinese Academy of Sciences, Wenzhou, Zhejiang 325000, China.

Contact information: [zhangyi@sinap.ac.cn](mailto:zhangyi@sinap.ac.cn) (ZY); E-mail: [hujun@sinap.ac.cn](mailto:hujun@sinap.ac.cn) (HJ)

**Table of Content**

[The oxidative species in the reaction system 1](#_Toc126837953)

[The absorbance curves of N_2_ NBs 2](#_Toc126837954)

[The redox potentials of water solutions with or without N_2_ NBs 2](#_Toc126837955)

[Size distribution of N_2_ NBs after degassing 2](#_Toc126837956)

[Antioxidation of the transformed ultra-small N_2_ NBs 3](#_Toc126837957)

[Antioxidation of the ultra-small O_2_ NBs 4](#_Toc126837958)

[The oxidation curve of ABTS in water containing N_2_ NBs 5](#_Toc126837959)

[The auto-oxidation curve of pyrogallol in water containing N_2_ NBs 6](#_Toc126837960)

[UV data 7](#_Toc126837961)

[The oxidation curve of TMB in water containing N_2_ NBs in the Fe^2+^/H_2_O_2_ system 7](#_Toc126837962)

[References 8](#_Toc126837963)

# The oxidative species in the reaction system

In this work, the radicals were generated by a Fenton-like reaction where hydroxyl radicals were the main intermediate production as indicated by the common oxidation indicator TMB. The ESR data (Figure S1) proved that the main radical was hydroxyl radicals rather than other radical species.


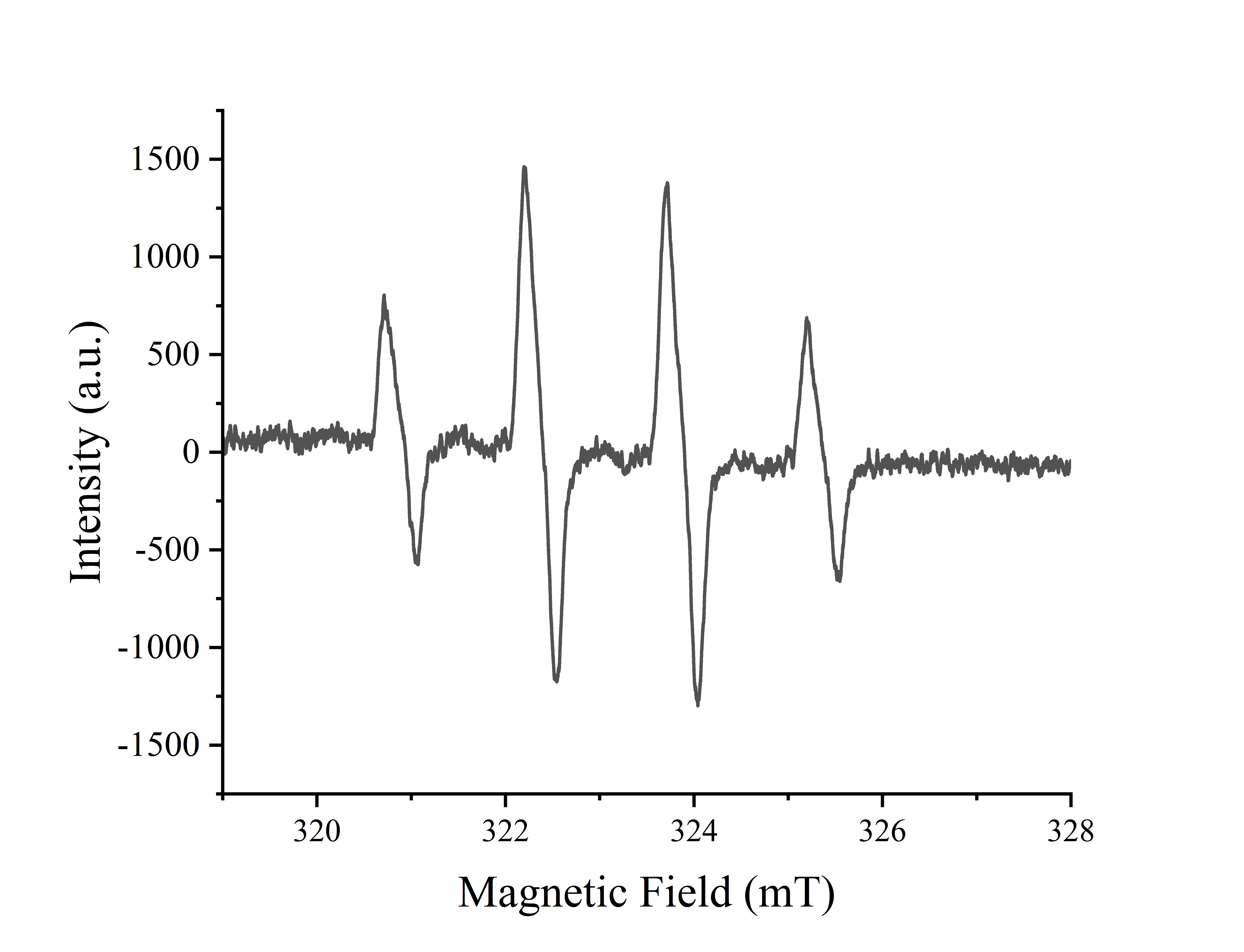


Figure S1. The ESR spectrum of the reaction system.

To prove that it was hydroxyl radicals but not other oxidative species such as Cu^2+^ or H_2_O_2_ in the reaction system that oxidized TMB, the oxidation curves of TMB in the presence of only H_2_O_2_ or Cu^2+^ were obtained. Results showed that there was no oxidation of TMB within 0.5 h (Figure S2), meaning that the oxidative properties of H_2_O_2_ or Cu^2+^ did not affect our conclusion.


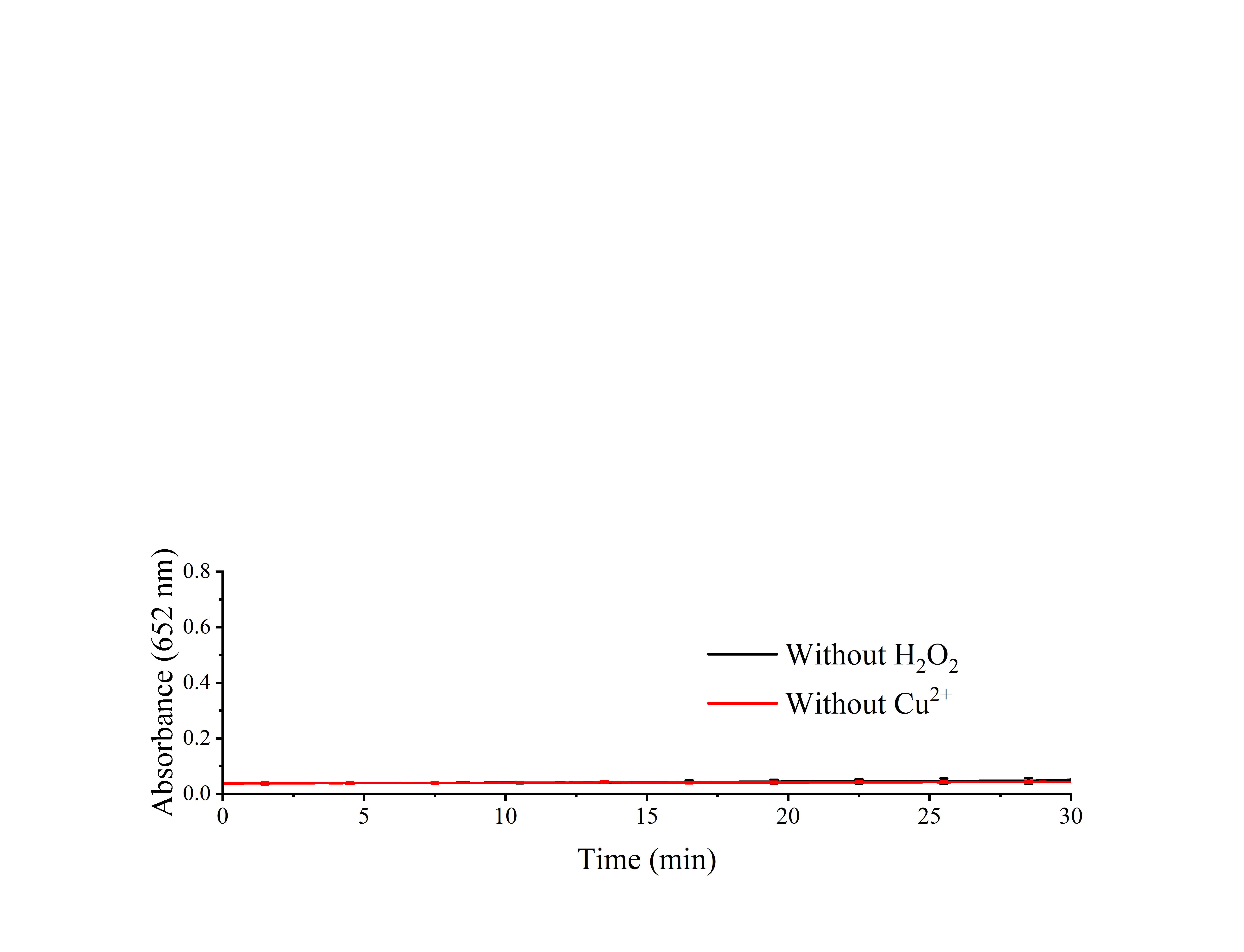


Figure S2. The oxidation curve of TMB without H_2_O_2_ or Cu^2+^.

# The absorbance curves of N_2_ NBs

The UV-VIS spectra of water containing normal N_2_ NBs or ultra-small N_2_ NBs were obtained. As shown in Figure S3, the absorption curves of N_2_ NBs seem almost identical to that of pure water, suggesting that the N_2_ NBs would not affect the absorbance of TMB at 652 nm.


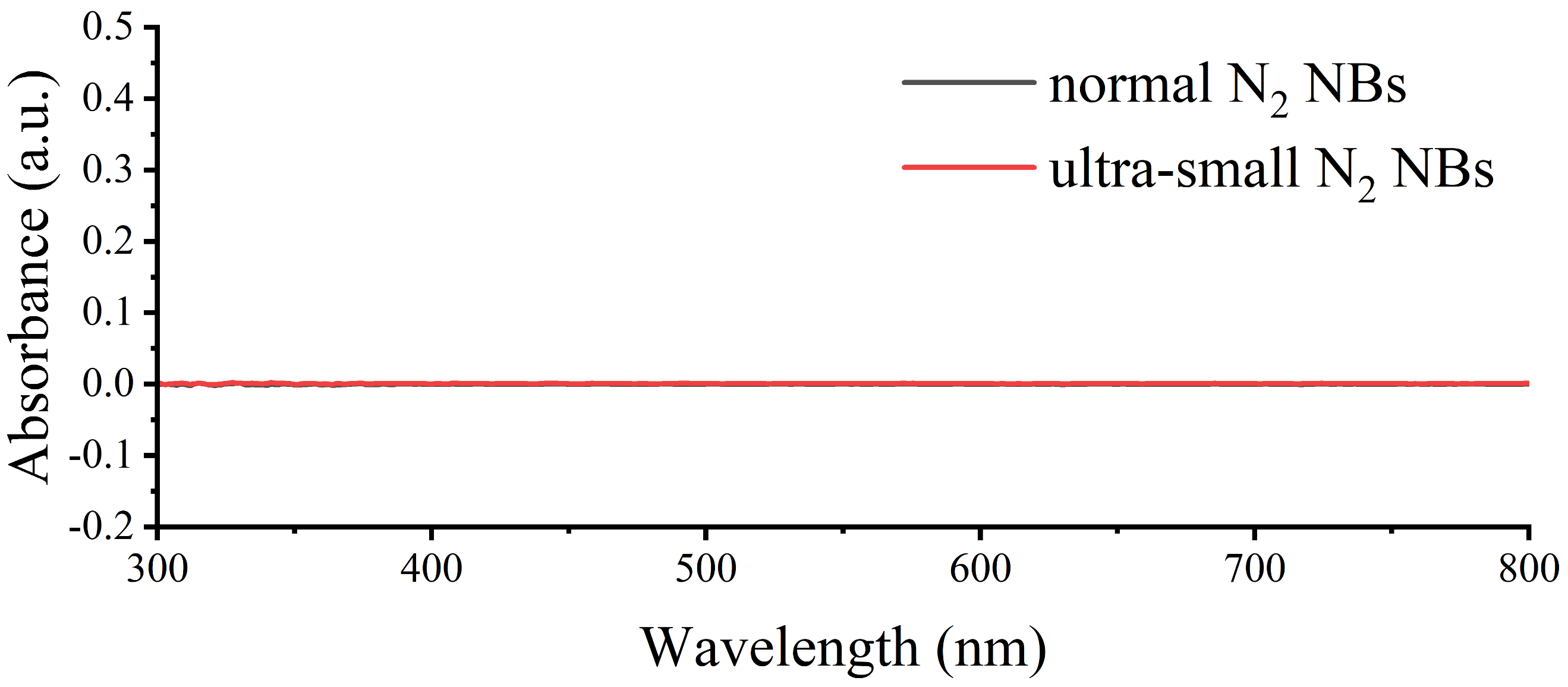


Figure S3. The absorbance curves of water containing normal N_2_ NBs or ultra-small N_2_ NBs. The spectrum of pure water was used as the baseline.

# The redox potentials of water solutions with or without N_2_ NBs

The redox potentials of pure water, normal N_2_ NBs, and ultra-small N_2_ NBs were exhibited in Table S1.

Table S1. The redox potentials of water solutions with or without NBs.

| **Items** | **redox potentials /*mV*** |
| --- | --- |
| Pure water | 249 |
| normal N_2_ NBs | 249 |
| ultra-small N_2_ NBs | 257 |

# Size distribution of N_2_ NBs after degassing

After the ultra-small N_2_ NBs were removed by degassing under reduced pressure, the NTA results (Figure S4) showed that the bubble concentration decreased from 5.42×10^7^ ± 5.78×10^6^ to 2.91×10^7^ ± 7.09×10^6^ particle/ml, and no N_2_ NBs smaller than 50 nm existed. The DLS results showed that the bubble concentration after degassing was below the detection limit, indicating that most of the N_2_ NBs smaller than 50 nm had been removed.


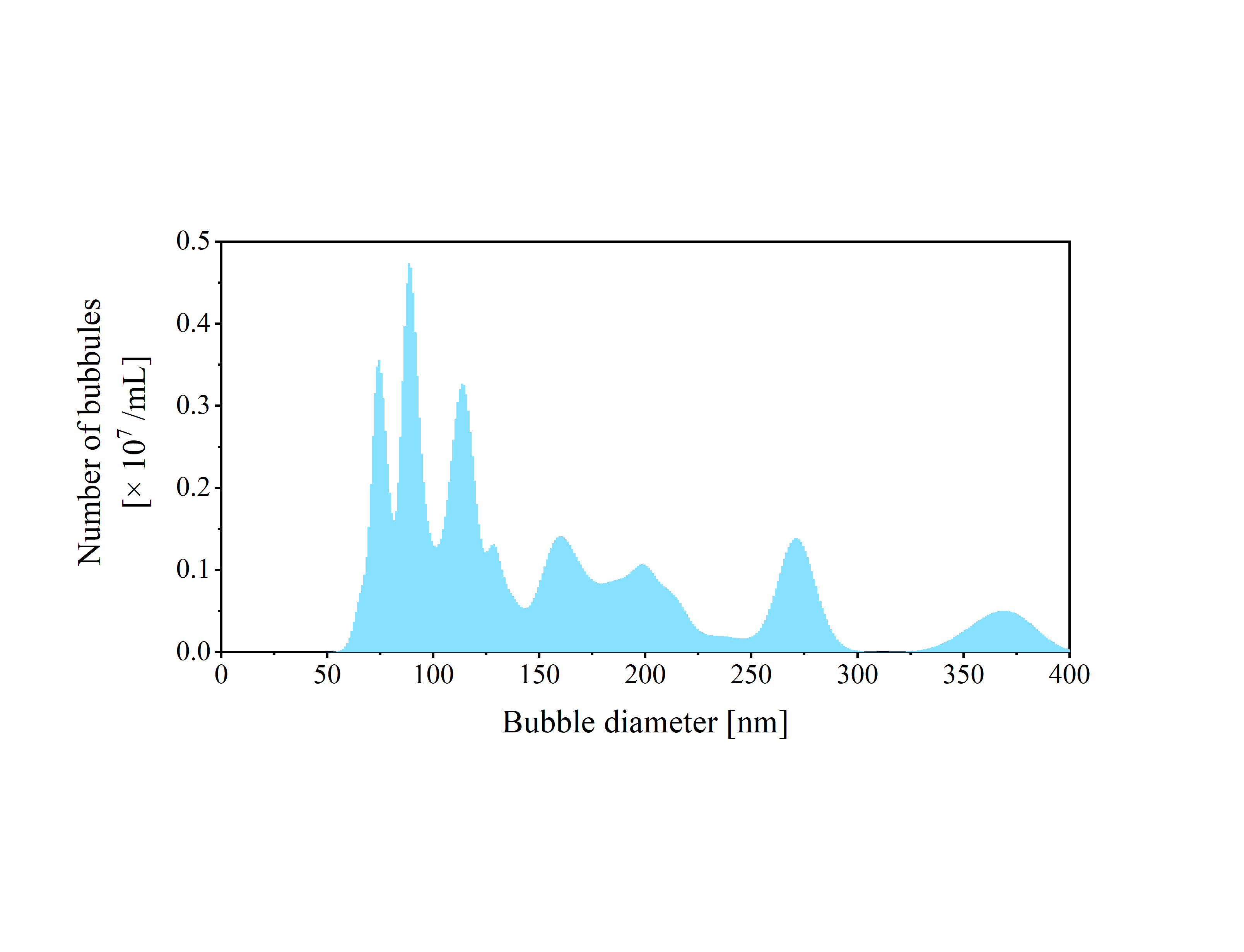


Figure S4. Size distribution of N_2_ NBs after degassing

# Antioxidation of the transformed ultra-small N_2_ NBs

Previous study^2^ has reported that in some cases, the normal NBs may be the clusters of ultra-small NBs, and the NB clusters could be dissociated into ultra-small NBs through a freeze-thawing operation. We suggest that the normal N_2_ NBs shown in Figure 2b might also contain clusters of ultra-small N_2_ NBs. Therefore, a freeze-thawing operation was conducted to generate smaller N_2_ NBs. As expected, the DLS study on the molten N_2_ NBs-containing water showed two peaks with strong scattering intensity at the NB sizes of 5.61 and 164 nm, respectively. That one peak centered at 5.61 nm observed in the DLS number percent curve (Figure S5-a) indeed suggests the dissociation of the large NBs into smaller ones. TMB oxidation curves also indicated that these dissociated N_2_ NBs could inhibit the oxidation of TMB (Figure S5-b).


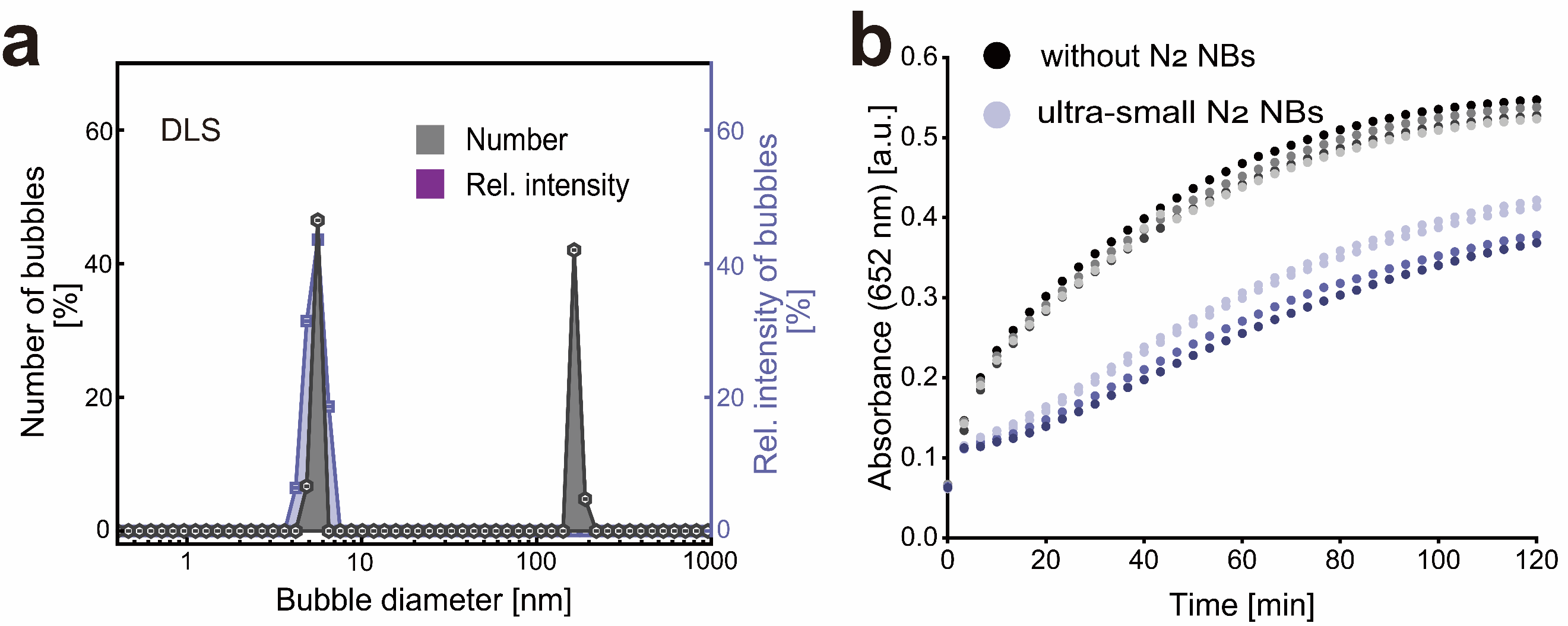


Figure S5. Antioxidation of the transformed ultra-small N_2_ NBs. (a) The light scattering intensity and particle number plotted versus the size of N_2_ NBs as measured by DLS after a freeze-thawing treatment of the N_2_ NB-containing water. (b) Oxidation curves of TMB in N_2_ NB-containing water after a freeze-thawing process.

# Antioxidation of the ultra-small O_2_ NBs

The ultra-small O_2_ NBs were prepared in cold ultrapure water (~0℃) by the compression−decompression method. Surprisingly, although O_2_ itself was a chemically oxidative reagent, oxidation curves (Figure S6-a) showed that the ultra-small O_2_ NBs also inhibited the oxidation of TMB by the hydroxyl radicals. NTA study showed a typical size distribution of the O_2_ NBs between 50-220 nm (Figure S6-b, upper), NB concentration of 4.23×10^7^ ± 5.66×10^6^ particle/ml, and an averaged NB size of 195.0 ±17.0 nm. DLS study revealed three strong scattering intensity peaks at NB sizes of 13.5 nm, 106 nm, and 295 nm, respectively (Figure S6-b, bottom). The antioxidation effect of the ultra-small O_2_ NBs implied that the cause of the antioxidant effect was most likely from the gas-liquid interfaces rather than the compositions of the gas molecules inside the NBs.


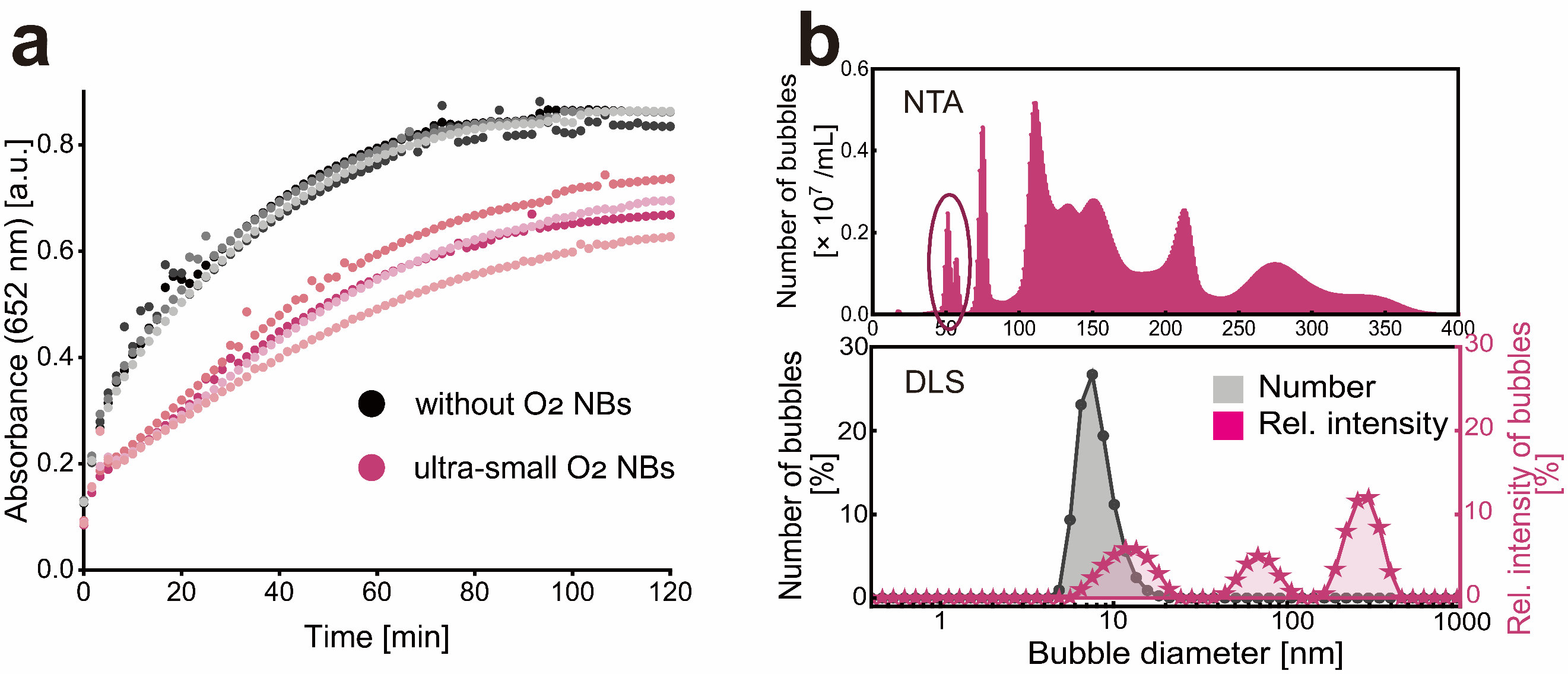


Figure S6. Antioxidation of the ultra-small O_2_ NBs. (a) Oxidation curves of TMB in water containing the ultra-small O_2_ NBs. (b) Size distribution curves of the ultra-small O_2_ NBs as measured by NTA (upper) and DLS (bottom). The circle highlighted the peaks of NBs with a size of about 50 nm.

# The oxidation curve of ABTS in water containing N_2_ NBs

To further study the antioxidating effect of NBs, ABTS, another classic •OH probe, was used instead of TMB in the reaction systems. The results (Figure S7) showed that normal N_2_ NBs enhanced slightly the oxidation of hydroxyl radicals, while ultra-small N_2_ NBs can inhibit the oxidation of hydroxyl radicals, agreeing well with that using TMB as the probe.


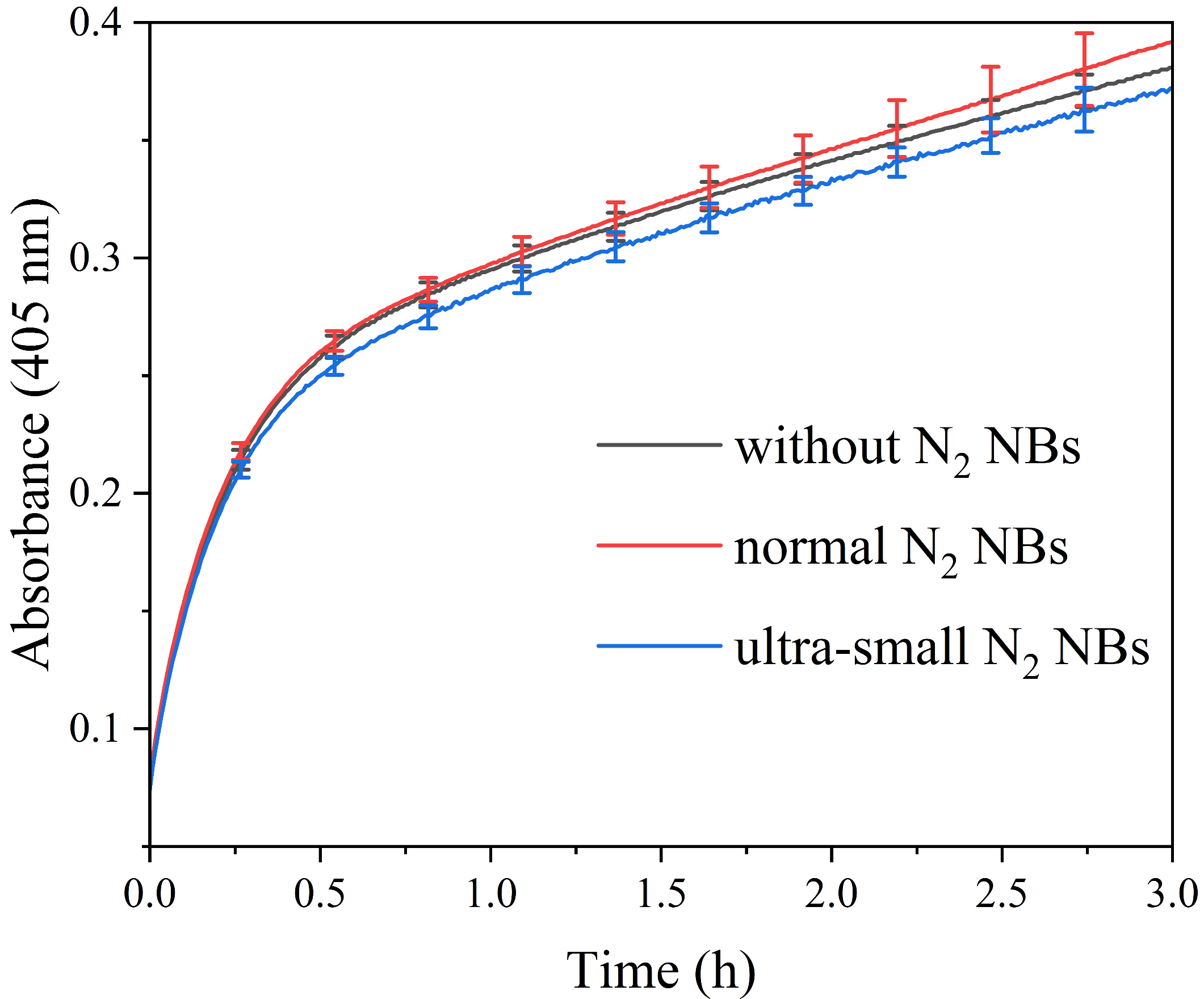


Figure S7. Oxidation curve of ABTS under normal N_2_ NBs or ultra-small N_2_ NBs.

# The auto-oxidation curve of pyrogallol in water containing N_2_ NBs

The effect of NBs on the superoxide anion radicals (O_2_^-^·) that have a longer half-life time than the OH· was tested. It is well-known that under alkaline conditions, pyrogallol can rapidly auto-oxidize to release O_2_^-^· and generate a colored intermediate product that has a strong light absorption at the wavelength of 325 nm. When there is a substance capable of quenching O_2_^-^·, the accumulation of the intermediate products would be prevented. As shown in Figure S8, when the ultra-small N_2_ NBs were presenting in the system, the absorbance at 325 nm on the autooxidation curve of pyrogallol was lower than those in water with or without normal N_2_ NBs at a fixed reaction time. The results indicate that ultra-small N_2_ NBs can effectively quench O_2_^-^·, while normal N_2_ NBs have no obvious effect.


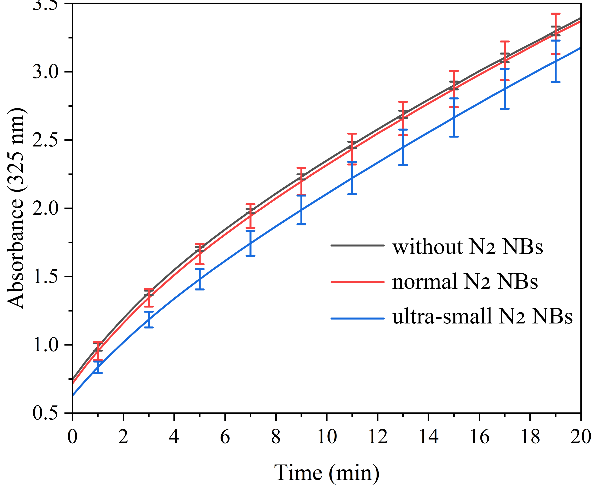


Figure S8. Autooxidation curve of pyrogallol under normal N_2_ NBs or ultra-small N_2_ NBs.

# UV data


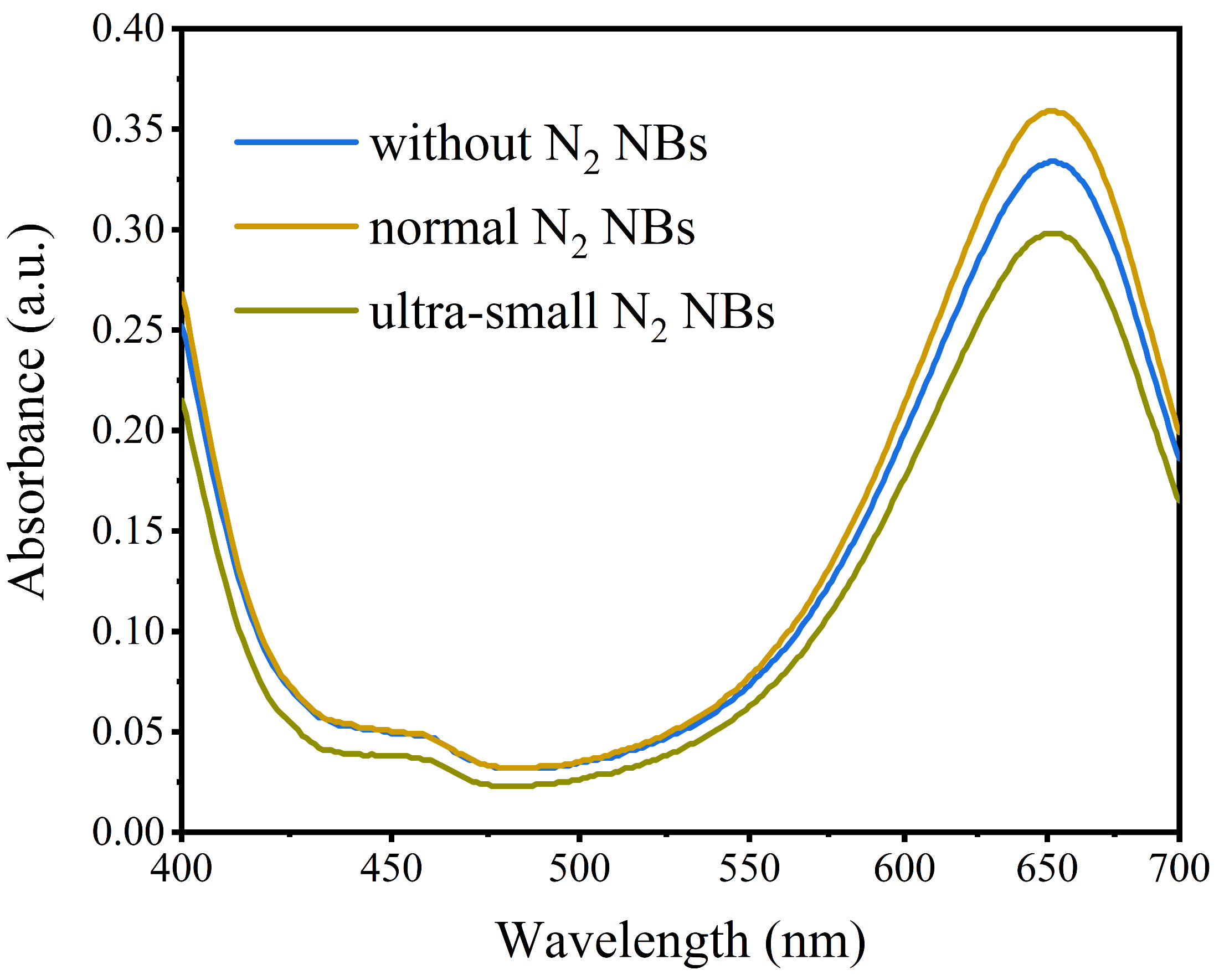


Figure S9. The UV absorbance curve of oxidized TMB (652 nm) in the reaction systems.

# The oxidation curve of TMB in water containing N_2_ NBs in the Fe^2+^/H_2_O_2_ system

To further verify the oxidation of ultra-small NBs, the more classical Fe^2+^/H_2_O_2_ was chosen to produce hydroxyl radical. A similar antioxidation effect of ultra-small N_2_ NBs has been found (Figure S10). However, in such a system the generation of hydroxyl radicals is very fast and hard to control. Besides, Fe^2+^ will be oxidized in the aqueous solution to form Fe^3+^, which will strongly interfere with our results. Therefore, we chose less reactive Cu^2+^ as catalysts to smoothly generate hydroxyl radicals.


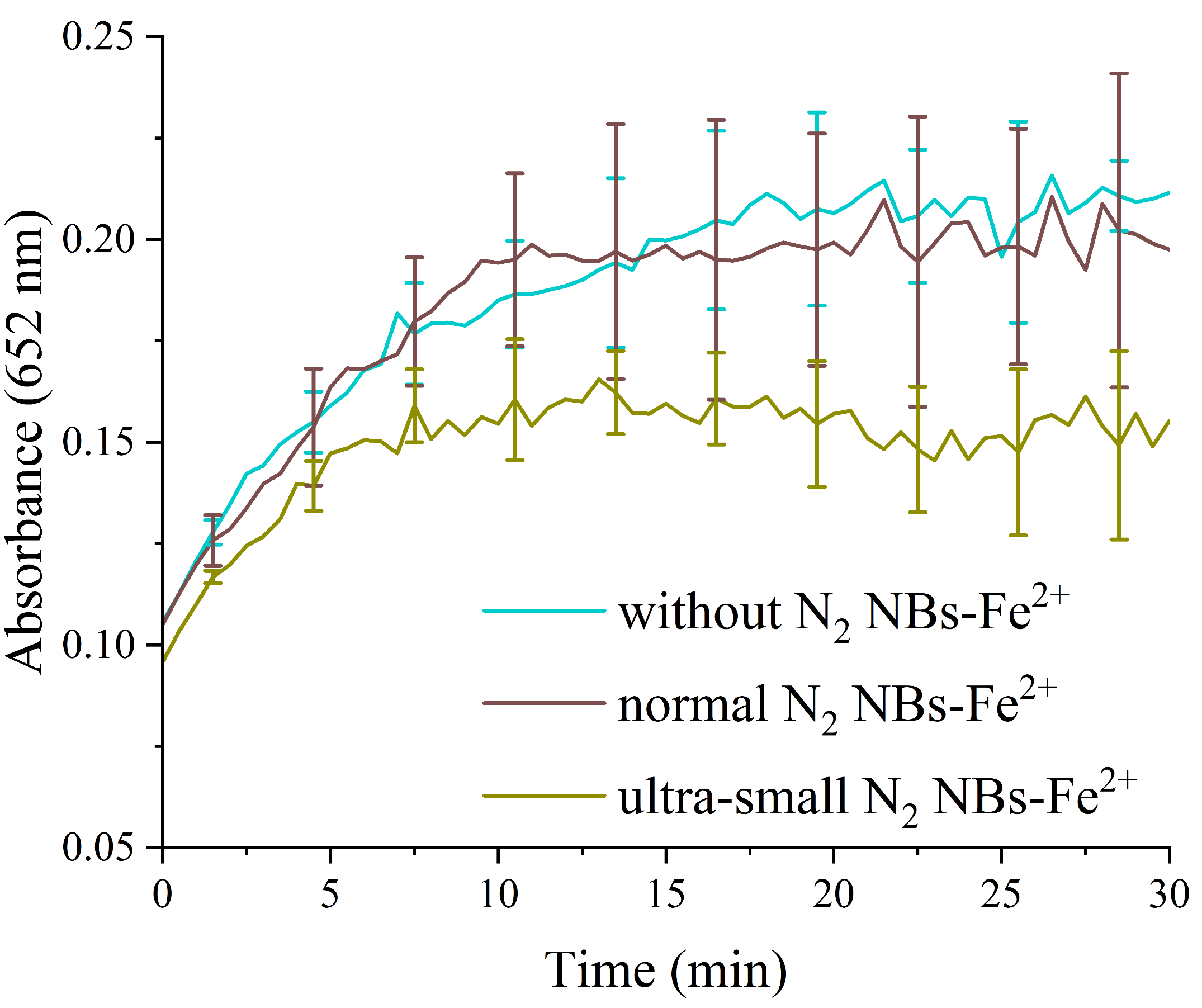


Figure S10. Oxidation curve of TMB under normal N_2_ NBs or ultra-small N_2_ NBs in Fe^2+^/H_2_O_2_ system.

# References

(1) Ke, S.; Xiao, W.; Quan, N.; Dong, Y.; Zhang, L.; Hu, J., Formation and stability of bulk nanobubbles in different solutions. *Langmuir* **2019,** *35* (15), 5250-5256.

(2) Jadhav, A. J.; Barigou, M., On the clustering of bulk nanobubbles and their colloidal stability. *J. Colloid Interface Sci.* **2021,** *601*, 816-824.

(3) Gong, C.; Li, D.; Li, X.; Zhang, D.; Xing, D.; Zhao, L.; Yuan, X.; Zhang, X., Spontaneous reduction-induced degradation of viologen compounds in water microdroplets and its inhibition by host–guest complexation. *J. Am. Chem. Soc.* **2022,** *144* (8), 3510-3516.
